# Earth’s youngest banded iron formation implies ferruginous conditions in the Early Cambrian ocean

**DOI:** 10.1038/s41598-018-28187-2

**Published:** 2018-07-02

**Authors:** Zhi-Quan Li, Lian-Chang Zhang, Chun-Ji Xue, Meng-Tian Zheng, Ming-Tian Zhu, Leslie J. Robbins, John F. Slack, Noah J. Planavsky, Kurt O. Konhauser

**Affiliations:** 1 State Key Laboratory of Geological Processes and Mineral Resources, China University of Geosciences, Beijing, 100083 China; 2 Key Laboratory of Mineral Resources, Institute of Geology and Geophysics, Chinese Academy of Sciences, Beijing, 100029 China; 3 Department of Earth and Atmospheric Sciences, University of Alberta, Edmonton, Alberta T6G 2E3 Canada; 4 U.S. Geological Survey, National Center, MS 954, Reston, Virginia 20192 USA; 5 Department of Geology and Geophysics, Yale University, New Haven, Connecticut 06520 USA

## Abstract

**It has been proposed that anoxic and iron-rich (ferruginous) marine conditions were common through most of Earth history**. **This view represents a major shift in our understanding of the evolution of marine chemistry**. **However**, **thus far**, **evidence for ferruginous conditions comes predominantly from Fe-speciation data**. **Given debate over these records**, **new evidence for Fe-rich marine conditions is a requisite if we are to shift our view regarding evolution of the marine redox landscape**. **Here we present strong evidence for ferruginous conditions by describing a suite of Fe-rich chemical sedimentary rocks—banded iron formation (BIF)—-deposited during the Early Cambrian in western China**. **Specifically**, **we provide new U-Pb geochronological data that confirm a depositional age of ca**. **527 Ma for this unit**, **as well as rare earth element (REE) data are consistent with anoxic deposition**. **Similar to many Algoma-type Precambrian iron formations**, **these Early Cambrian sediments precipitated in a back-arc rift basin setting**, **where hydrothermally sourced iron drove the deposition of a BIF-like protolith**, **the youngest ever reported of regional extent without direct links to volcanogenic massive sulphide (VMS) deposits**. **Their presence indicates that marine environments were still characterized by chemical- and redox-stratification**, **thus supporting the view that—despite a dearth of modern marine analogues—ferruginous conditions continued to locally be a feature of early Phanerozoic seawater**.

## Introduction

It is well established that the Archaean and Palaeoproterozoic Earth was characterized by anoxic, ferruginous oceans, as evidenced by the deposition, and subsequent cessation of, large-scale BIFs^1,2^. Traditionally, the oceans were envisioned to have become predominantly oxic^3^ or euxinic^4,5^ around 1.8 Ga, coincident with deposition of the last economic iron formation. These traditional views were challenged, and it was later suggested that ferruginous conditions were a common feature after the end of BIF deposition^6^. In a series of seminal papers, Canfield and colleagues proposed that ferruginous conditions were common in the mid-Ediacaran (580–560 Ma)^7^. It was subsequently proposed that ferruginous conditions were prevalent during Earth’s middle history^8,9^, and even into the Cambrian^10^ during the early diversification of animals and establishment of trophically complex ecosystems^11^.

Current evidence for ferruginous conditions in the Cambrian comes mainly from Fe-speciation data for marine shales, a calibrated proxy that relies on linking ratios of biogeochemically active iron phases, such as sulphides, carbonates, and oxides, to water-column redox conditions^8,12^. However, Fe-speciation signatures can be ambiguous. Critically, a range of modern marine settings, foremost sediments deposited in relatively near-shore settings, display Fe-speciation patterns equivalent to those linked to ferruginous conditions^13–17^. Moreover, detailed petrographic work has suggested that common diagenetic processes can lead to ambiguous Fe-speciation signatures and specifically false signals of ferruginous coniditons^18^. Numerous studies have drawn on alternative methods for tracking ocean anoxia in the Cambrian, including the use of redox-sensitive trace element enrichments or carbon and sulphur stable isotopes^19–21^. Additionally, non-traditional stable isotopes, such as those for uranium^22^ or molybdenum^23,24^, have been used to directly track euxinia in the Cambrian, but critically, not ferruginous conditions. Although anoxia is a prerequisite to euxinia, strong euxinic conditions develop as the result of abundant organic carbon export and strong bacterial sulphate reduction driving elevated sulphide concentrations. However, these conditions are unlikely to be representative of background marine environments^8^.

Similar to what is observed in the Archaean and periodically through the rest of the Precambrian^1,2^, the presence of iron-rich chemical sedimentary rocks (i.e., BIFs), can provide a clear signature of ferruginous conditions. However, until now, there have been no extensive, basin-scale marine Fe-rich chemical sedimentary rocks reported from the Lower Cambrian that would be indicative of widespread ferruginous conditions during this critical transition in Earth history. In this paper, we couple geochronological data with mineralogy, sedimentary features, and REE systematics from a suite of Fe-rich chemical sedimentary rocks in the Taxkorgan terrane, western China, and provide strong evidence for ferruginous conditions in the Early Cambrian. Importantly, these sedimentary rocks closely resemble Archaean Algoma-type BIF, sedimentary rocks that formed close to volcanic arcs and spreading centres, having been produced by exhalative hydrothermal processes related to submarine volcanism in either partially closed basins or open seawater systems^25^. As such, our newly described BIFs are the only regionally extensive examples of this type thus far reported from the Phanerozoic, without having spatial and stratigraphic links to known VMS deposits.

## Results

### Sedimentary features and mineralogy

**Figure 1:**
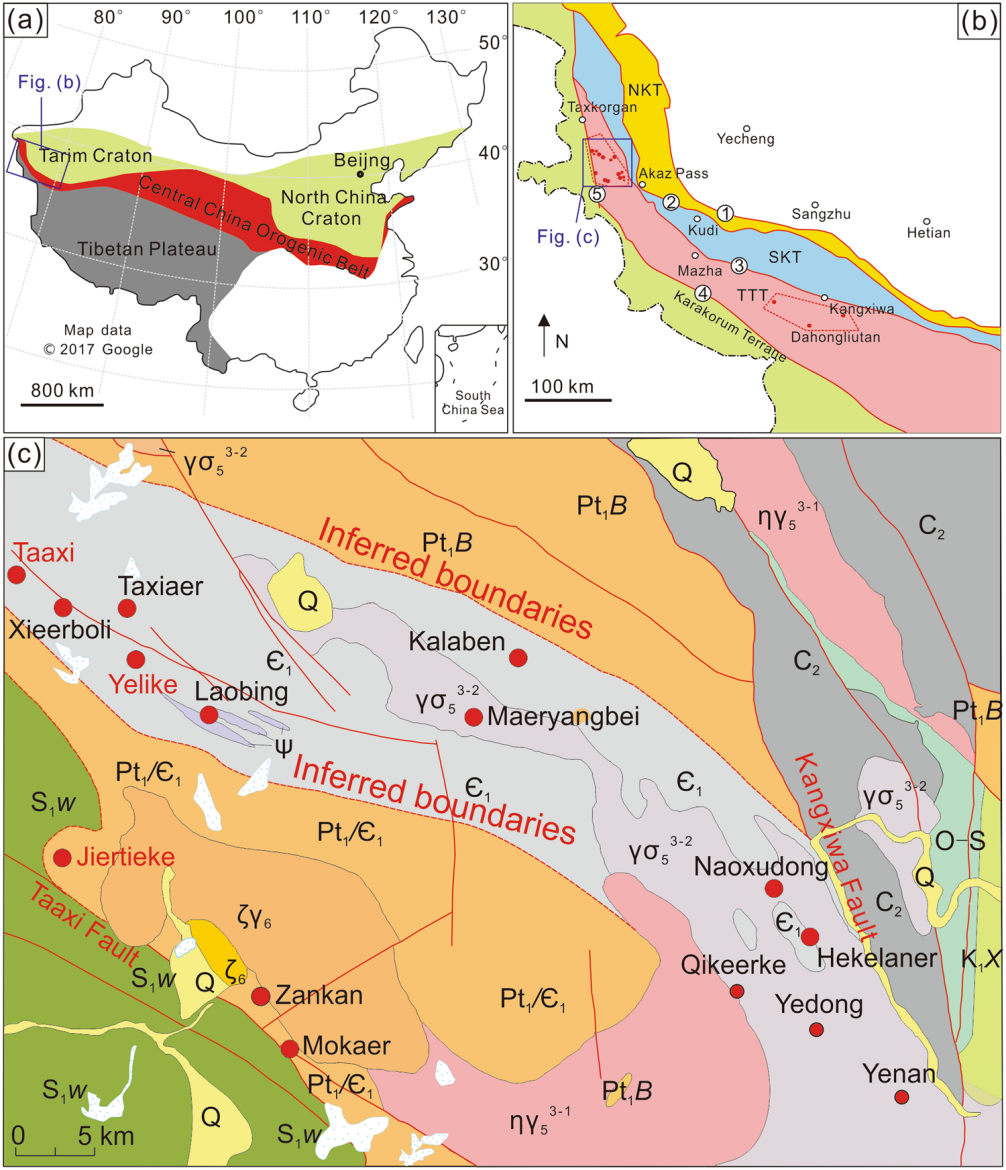
(**a**) Tectonic subdivisions of China and location of Western Kunlun orogenic belt; (**b**) Tectonic subdivisions of West Kunlun orogenic belt, showing Taxkorgan region, location of Fe deposits and basins are marked by red dots and dashed lines, respectively. (**c**) Geological map and distribution of metasedimentary Fe deposits in south Taxkorgan area; Legend: NKT: North Kunlun Terrane, SKT: South Kunlun Terrane, TTT: Taxkorgan-Tianshuihai Terrane, ①: Oytag-Kegang Fault, ②: Kudi Fault, ③: Kangxiwa Fault, ④: Longmucuo-Shuanghu Fault, ⑤: Karakorum Fault; Q: Quaternary, K_1_X: Cretaceous, C_2_: late Carboniferous, S_1_W: early Silurian, O-S: Ordovician-Silurian, Є_1_: Early Cambrian, Pt_1_/Є_1_: Early Proterozoic or Early Cambrian, Pt_1_B: Early Proterozoic, ζ_6_: Himalayan syenite, ζγ_6_: Himalayan syenogranite, γσ_5_^3–2^: Yanshanian granodiorite, ηγ_5_^3-1^: Yanshanian monzonitic granite, ψ: Pyroxenite, red spots are locations of iron-rich sediments. Figure 1a was made with data from Google Maps (https://www.google.com/maps), and reproduced using CorelDRAW x7 (http://www.coreldraw.com/en/). Figure 1b,c were after refs^26,27^.

The Taxkorgan terrane occurs within the West Kunlun orogenic belt, southwest of the Tarim block. The region is dominated by the Bulunkuole (Pt_1_) and Bulunkuole (Є_1_) Groups (Fig. 1a,b)^26,27^. Strata of the Bulunkuole Group are monoclinal, striking 358°−45°, with moderate to steep dips ranging from 20° to 75°. The strata consist of metasedimentary rocks (e.g., biotite-quartz schist), metavolcanic rocks (e.g., metabasalt, metadacite), Fe-rich sedimentary rocks, and dolomitic marble that experienced greenschist- to amphibolites-facies metamorphism during the Caledonian orogeny (492–428 Ma)^28^. Corresponding protoliths were most likely greywacke, volcaniclastic rocks, bimodal volcanics (dacite and basalt), iron formation, and carbonates. A description of the regional geology is provided in the Supplementary Information. The Fe-rich chemical sediments mainly occur in the Jiertieke, Yelike, Taaxi, Zankan, and Laobing areas (Fig. 1c). All of these strata trend NW to SE and formed as part of an extensive belt that contains more than 500 Mt of known iron ore, with a prospective aggregate size reaching 1 Gt. We limited our sampling to the Jiertieke, Yelike, and Taaxi areas because the strata there did not experience significant post-depositional alteration (i.e., fewer veins, fracture zones, and intrusions), and are compositionally analogous to typical Precambrian BIF. In terms of areal extent, these three Fe-rich deposits have surface dimensions of 31 km^2^, 18 km^2^, and 8 km^2^ for the Yelike, Jiertieke, and Taaxi areas, respectively. Collectively, these three main areas preserve over 90 Mt of iron ore.

**Figure 2:**
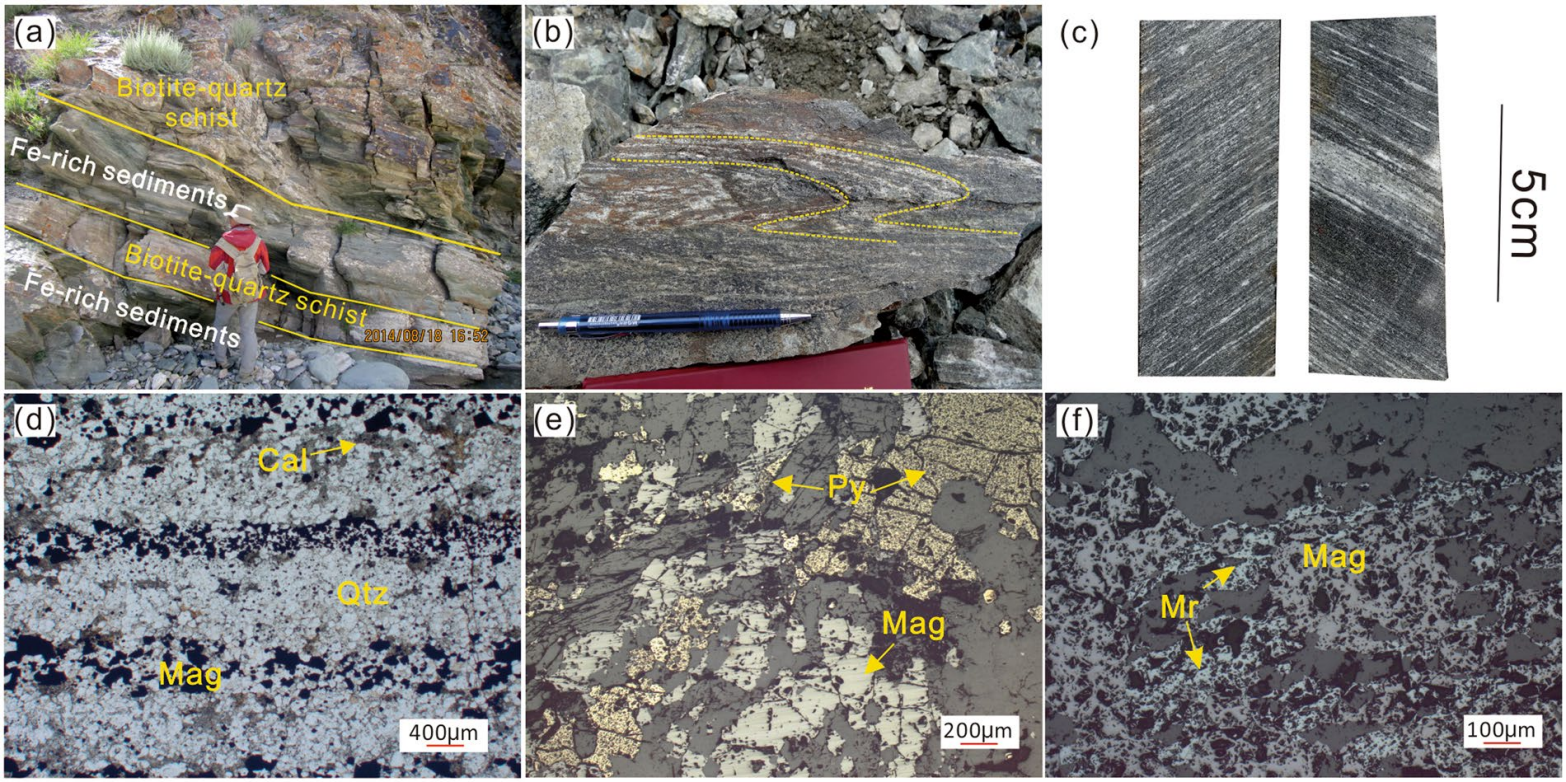
(**a**) Well-bedded biotite-quartz schist alternating with BIF; (**b**) “S-type” folds in BIF retain initial banded structure; (**c**,**d**) BIF shows characteristic Fe-rich (dark) and Si-rich (light) layers; (**e**) Pyrite replaces euhedral magnetite crystal near a fracture zone; (**f**) Martite replacement of magnetite grain. Abbreviation: Mag-magnetite, Qtz-quartz, Cal-calcite, Py-pyrite, Mr-martite.

The Fe-rich chemical sediments display laminated and lenticular structures and are typically interlayered with metasedimentary rocks (biotite-quartz schist) in outcrop, and locally are in contact with meta-volcaniclastic rocks and dolomitic marble in drill core. Rocks below the Fe-rich chemical sedimentary rocks are dominated by metavolcanics (30 m thick) and dolomitic marble (>50 m). The main host rock, biotite-quartz schist, has a medium- to fine-grained lepidoblastic texture, as well as schistose and banded structures. This schist is well bedded, and alternates with the Fe-rich sedimentary rocks (Fig. 2a), displaying typical sedimentary features. Ratios of immobile trace elements in the biotite-quartz schist (Nb/Y; Ti/Zr, Fig. S1) are similar to those of metadacites in the region, suggesting that the schist has a major felsic volcanic component, consistent with Th/Sc ratios of 1.07–1.13 (Table S1). Although the BIF locally shows “S-type” and other folds, primary banding structures are preserved (Fig. 2b). Constituents are predominantly quartz (~60%) and lesser biotite (~20%), with minor chlorite (~12%) and magnetite (~3%). The biotite tends to be fine grained, 0.3–1.2 mm in size, and locally replaces chlorite.

The Fe-rich sedimentary rocks are characterized by Fe-rich layers, composed of magnetite, and Si-rich layers, composed mostly of quartz, with lesser calcite, biotite, and magnetite (Fig. 2c,d). Bulk compositions are dominated by iron (FeO 29.03–77.8 wt.%) and silica (SiO_2_ 18.0–56.3 wt.%) (Table S2); BIFs are compositionally defined as having a Fe concentration within this range (i.e., at least 15 wt.% Fe)^29^. Magnetite is anhedral, and locally together with quartz, displays a blastopsammitic texture, including similar grain sizes and a clear paragenetic link. Martite is common within the oxidized ores and locally replaces magnetite crystals in the BIF (Fig. 2e). No pyrite was found within the BIF; the only pyrite observed is near fracture zones (Fig. 2f), characteristic of a secondary origin.

### Geochronology of the Jiertieke area

Previous studies on the age of the Bulunkuole Group have suggested either a Palaeoproterozoic^30^ or early Paleozoic age^31–33^, and until now the depositional age has remained unresolved. Palaeoproterozoic zircons can normally be found within the southern section of the Bulunkuole Group near the Zankan iron deposit, whereas early Paleozoic zircons occur in several different locations^27,34^. Zhang *et al*.^26^ suggested that these contrasting zircon ages may be reconciled by dividing the Bulunkuole Group into two parts: (1) Palaeoproterozoic strata in the south, which include the Zankan and Mokaer areas, and (2) Cambrian strata in the Yelike, Taaxi, and Laobing areas that extend from the southwest to the northeast. The Jiertieke area, studied here, lies between Taaxi-Laobing and Zankan-Mokaer. Knowing the exact age of the Jiertieke BIF is, therefore, critical for resolving the age and genesis of this deposit.

**Figure 3:**
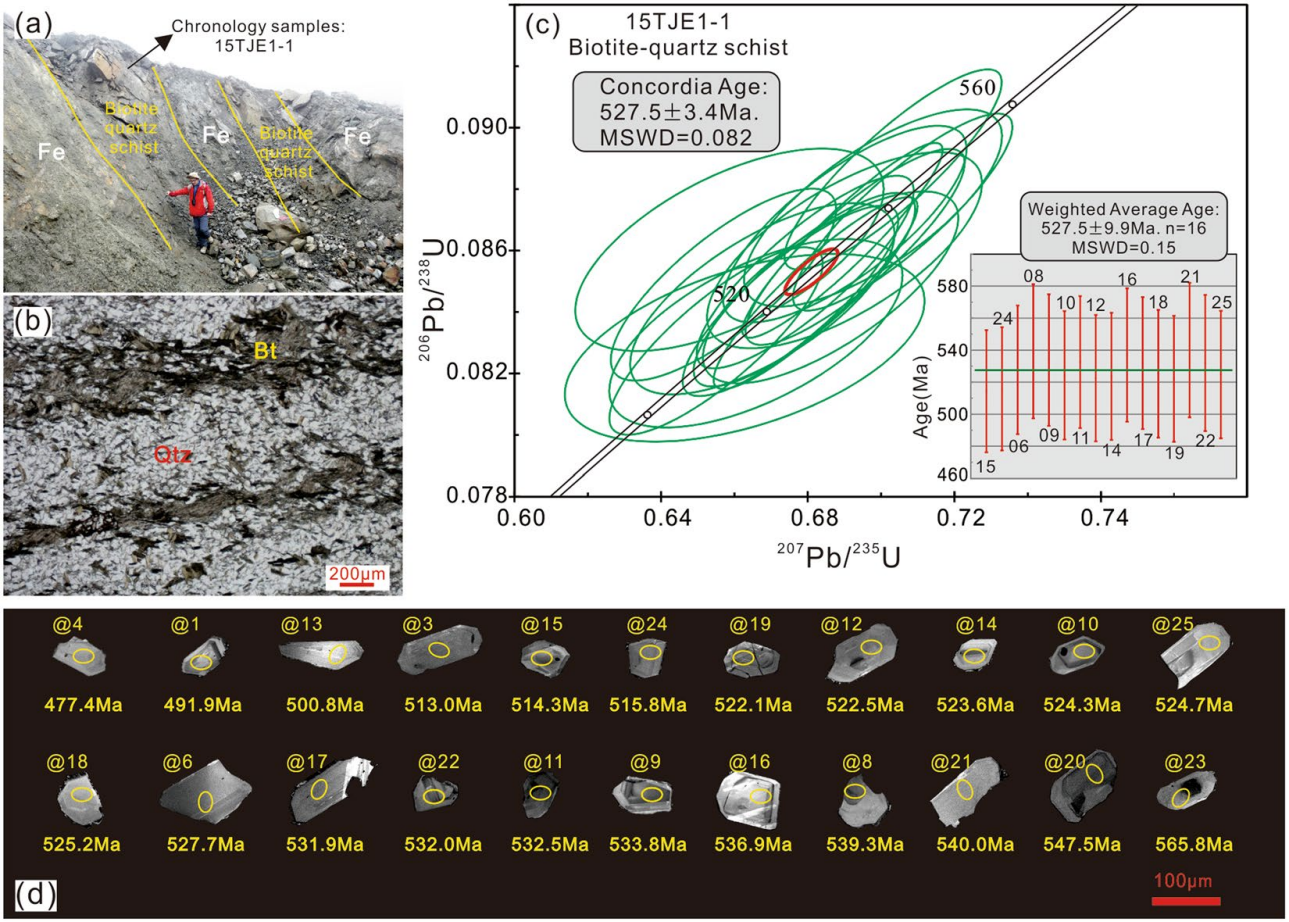
(**a**) Outcrop photo showing location of samples used for geochronological analyses; (**b**) thin section photo in plane polarized light of biotite-quartz schist; (**c**) Concordia plot and weighted average plot for zircon U-Pb ages from biotite-quartz schist; (**d**) SEM cathodoluminescence images with corresponding apparent ages of zircons from biotite-quartz schist; note lack of rounded grains.

We analyzed U-Pb isotopes in zircon to constrain the age of the BIF, which previously was interpreted as either Cambrian or Palaeoproterozoic^26^. BIFs in the Jiertieke area are conformable, stratiform, and interbedded with biotite-quartz schist. Therefore, the schist can used to constrain the formational age of the BIF. The location of the biotite-quartz schist sample 15TJE1-1, from which zircons were obtained, is shown in Fig. 3. Many of the zircons are euhedral to subhedral, 100–140 μm in length, with large length-to-width ratios (up to 3). Concentric zoning is common in these zircons, sharing typical features of zircons crystallized from granitic magma. The sharp edges likely reflect short-distance transport, potentially from the metadacite in the region (see above). Most of the zircons lack overgrowth rims (Fig. 3), suggesting that late-stage processes, such as metamorphism, had minimal if any post-crystallization effects. *In situ* spot analyses for U, Th, and Pb were conducted on 22 subhedral to euhedral zircon grains, which have variable Th and U contents with Th/U ratios ranging from 0.57–1.11, suggesting an origin as magmatic zircon. Most grains yielded ^206^Pb/^238^U ages between 547.5 to 513 Ma, with a Concordia Age of 527.5 ± 3.4 Ma (MSWD = 0.082) (see Supplementary Table S3), which is similar to the weighted average age (*n* = 16) of 527.5 ± 9.9 Ma (MSWD = 0.15). These ages correspond with those of the Taaxi metadacite (553–531 Ma; 524–520 Ma)^27,34^, suggesting that the zircon grains are derived from the coeval dacite in the region (Fig. 3). Moreover, a recent detailed geochronological study of U-Pb ages of zircons from a granitic intrusion in the central section of the Bulunkuole Group (close to Jiertieke), yielded an age of 510–513 Ma^35^, which can be used to constrain the lower limit of the formational age. Given this evidence for an Early Cambrian depositional age, the critical question becomes how did the BIFs precipitate, and crucially, what information can they provide about Early Cambrian redox conditions?

### Major and trace elements of the iron-rich chemical sediments

In addition to FeO and SiO_2_, other oxides present above trace levels include CaO (0.41–15.2 wt.%), MgO (0.11–7.16 wt.%), MnO (0.01–1.05 wt.%), K_2_O (0.00–0.65 wt.%), TiO_2_ (0.01–0.28 wt.%), and P_2_O_5_ (0.03–0.16 wt.%). Locally high Mn concentrations suggest that a significant flux of Mn(II) was brought into the basin, a condition consistent with, but not entirely dependent upon, anoxia in the deep part of the water column^36^. The BIFs from Jiertieke and Taaxi also have lower Al_2_O_3_ and Na_2_O contents (Al_2_O_3_ = 0.09–1.48 wt.%; Na_2_O = 0.02–0.74 wt.%) compared to Yelike (Al_2_O_3_ = 4.80–5.55 wt.%; Na_2_O = 1.67–3.01 wt.%). The important point here is that, although the major-element compositions indicate some detrital input to the BIF, the Fe-rich sedimentary rocks are predominantly chemical in origin.

Rare earth element data are one of the most commonly used geochemical tools to understand the origin of chemical sedimentary rocks such as BIF^37–41^. In particular, REE patterns can indicate whether the Fe-rich sediments formed in, or near, the chemocline in a redox-stratified ferruginous basin. Further, the REE system is typically rock-buffered during the diagenesis and metamorphic alteration of chemical precipitates^42–44^, and thus is a powerful tool to track water-column redox conditions coeval with deposition^39,45,46^. Multiple aspects of the REE system are directly (Ce anomalies) or indirectly (light to heavy REE ratios, Y/Ho ratios) sensitive to redox conditions. Cerium can be oxidized from its trivalent to tetravalent state, which results in a large decrease in solubility; however, below the redoxcline, Ce can become reductively recycled, and negative Ce anomalies indicative of oxygenic conditions may disappear^47^. Additionally, owing to differences in particle reactivity in seawater, light REE depletion and large positive Y anomalies develop in oxic seawater. Within anoxic seawater, dissolution of oxides results in the suppression of Y anomalies and a shift to flat or even light REE-enriched patterns^41^.

**Figure 4:**
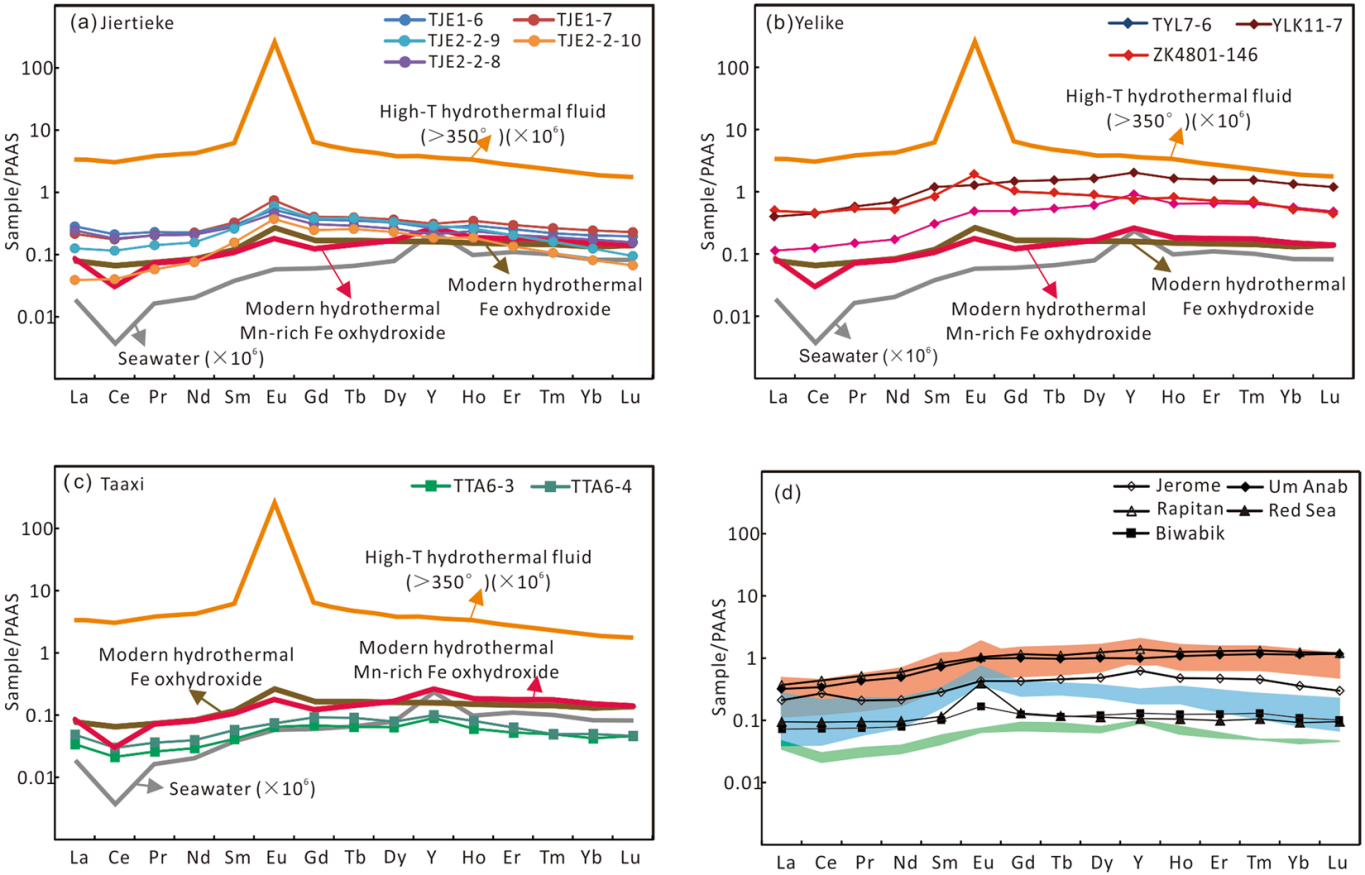
REE systematics of the BIF from (**a**) Jiertieke, (**b**) Yelike, and (**c**) Taaxi regions. Average South Pacific seawater, average high-T (>350 °C) hydrothermal fluid^70^, as well as modern oxic hydrothermal Fe-oxyhydroxide and Mn-rich Fe-oxyhydroxide samples^49^ are plotted for comparison. (**d**) Taxkorgan Fe-rich sediments relative to averages from several Palaeoproterozoic and Neoproterozoic BIF, including 1.74 Ga Jerome Formation (U.S.A.)^46^, 1.88 Ga Biwabik BIF (Animikie basin, U.S.A.)^71^, 715 Ma Rapitan Iron Formation (Canada)^72^, and 750 Ma Um Anab Formation (Egypt)^73^, as well as modern Fe-rich sediments from Atlantis II Deep (Red Sea)^74^. Samples from Jiertieke (blue), Yelike (red), and Taaxi (green) regions are highlighted. All data are normalized to PAAS values after McLennan^75^.

REE patterns in iron oxide-rich sedimentary rocks have been extensively used to track marine redox conditions^1,2,41^. Although iron oxides do not perfectly capture dissolved REE patterns in experiments^45^, hydrous iron oxides that precipitated in the water column can record patterns of dissolved REE in seawater e.g. ref.^48^). Similarly, although iron oxides precipitated at low pH can develop strong positive Ce anomalies^45^, these conditions are not relevant to marine environments distal from hydrothermal vents. As a modern example of the utility of REE patterns in iron oxides, their distribution at Loihi Seamount trace the shift from deposition within low-oxygen, iron-rich waters to fully oxic conditions^49^ (Fig. 4). Accordingly, iron oxides forming in low-oxygen waters (e.g., at chemocline of redox-stratified basins) should have a small or positive Ce anomaly, and less-pronounced light REE depletion and Y anomalies, than would be expected for oxic seawater. Taking this into account, REE can be used to track the presence of ferruginous waters in a basin. Some fractionation of REE among various mineral phases may result from diagenesis and metamorphism, but critically, the REE patterns of Fe minerals (e.g., magnetite and hematite) tend to correspond well to bulk-rock patterns^50^. Furthermore, bulk digestion methods, such as those employed here, yield seawater-like REE patterns, thus providing confidence that an authigenic signal is being preserved^41,51–53^.

**Figure 5:**
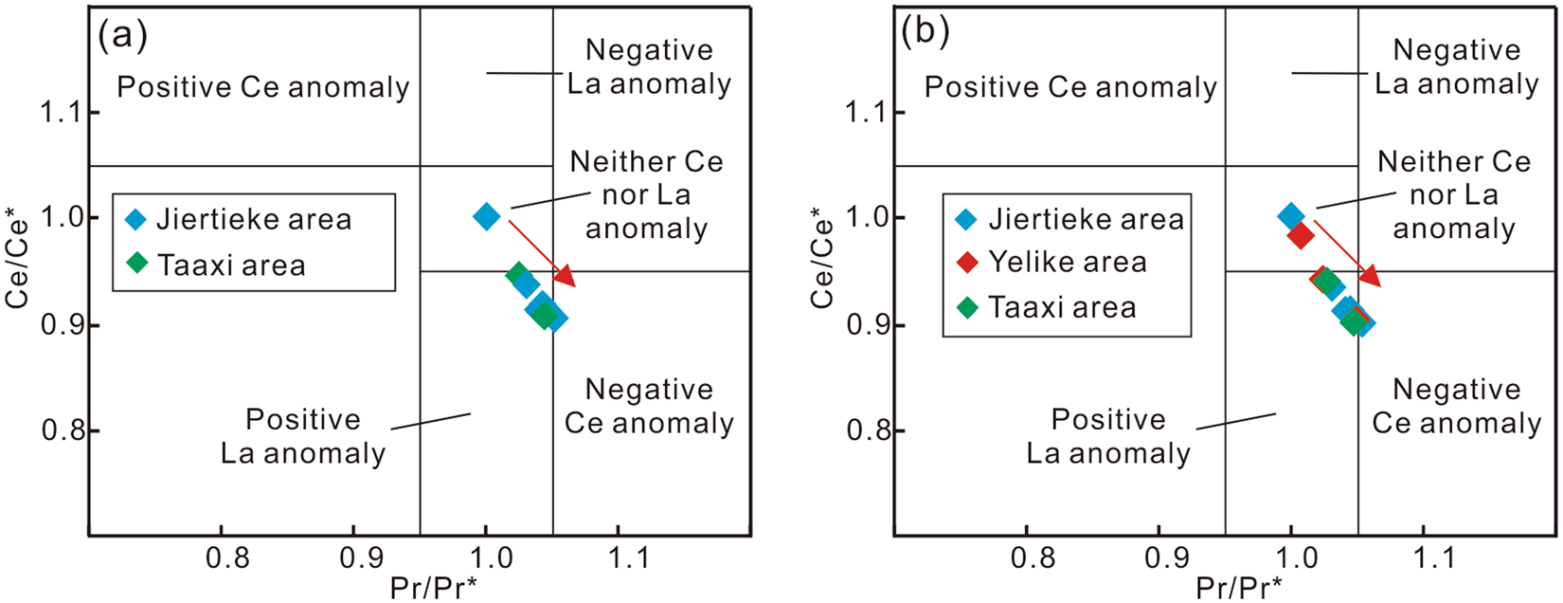
(**a**) Ce/Ce*-Pr/Pr* cross-plot indicates there are no true negative Ce anomalies for Taxkorgan Fe-rich sediments. Samples from Yelike region are excluded due to suspected detrital contamination; (**b**) Recalculated detrital-free basis using PAAS as detrital component and Hf as normalizing element (Table S4), lacks true negative Ce anomalies like the uncorrected bulk samples. Ce/Ce* was calculated as Ce/Ce* = Ce_PAAS_/(2Pr_PAAS_ − Nd_PAAS_), Pr/Pr* was calculated as Pr/Pr* = 2Pr_PAAS_/(Ce_PAAS_ + Nd_PAAS_), after Bolhar *et al*.^57^.

Iron-rich sedimentary rocks from the Yelike and Taaxi areas lack significant negative Ce anomalies (Fig. 5), but display features consistent with an authigenic, rather than detrital, origin of the REE. These features include positive Y anomalies (1.25–1.46), slight light REE depletion (La/Yb_PAAS_ = 0.20–0.97), and bulge among the middle REE (Fig. 4). As such, the REE patterns presented here are unlikely to reflect detrital signatures. Moreover, the above-mentioned features are indicative of authigenic REE sorption to iron oxides at, or near, a chemocline. Given the absence of negative Ce anomalies, the adsorption of REE likely took place within anoxic, rather than fully oxic, waters^41,45,54^. The REE patterns, together with the presence of magnetite-quartz layers and lack of associated VMS deposits (see below), suggests that the depositional style of the Early Cambrian Bulunkuole Group is broadly analogous to that of Precambrian BIFs, rather than of distal hydrothermal deposits that formed within an oxic ocean basin^41,55,56^. Samples from the Yelike and Jiertieke regions similarly record authigenic REE signatures (e.g., light REE depletion and variable Y/Ho ratios), and a lack of negative Ce anomalies.

Bolhar *et al*.^57^ suggested that terrestrial material (i.e., felsic and basaltic crust) is characterized by a near-constant Y/Ho ratio of ~26. As a consequence, admixtures of any detrital terrestrial contaminant should be evident through co-variation of Y/Ho ratios and detrital contaminants such as Al_2_O_3_ and Zr. However, only a weak correlation is apparent between Zr-Y/Ho and Al_2_O_3_-Y/Ho, as well as correlations with ∑REE + Y and Y/Y* (Fig. S2), thus effectively ruling out a detrital control on the Y/Ho ratios and REE patterns. Consistent with this view, near-chondritic Y/Ho ratios are found in samples having the lowest detrital input (e.g., TJE2-2-9, TJE2-2-10; indicated by low Al_2_O_3,_ TiO_2_, and Zr contents; Table S2). Moreover, REE data calculated on a detrital-free basis (using PAAS as the detrital component and Hf as the normalizing element) display the same REE patterns, Ce and Eu anomalies (Table S4, Ce/Ce* = 0.90–1.00; Eu/Eu* = 0.98–2.15) as the uncorrected bulk samples (Fig. S3). This supports that REE signatures are not controlled by detrital overprinting. Thus, the presented REE data point to primary iron oxyhydroxide particles deposited from a predominantly anoxic water column, with a loss of Y occurring below the chemocline, thereby explaining the concurrent absence of a negative Ce anomaly and depressed Y/Ho ratios (Fig. 4).

## Discussion and Conclusions

Previous research has argued that the West Kunlun orogenic belt, relevant to the Tarim block^58,59^ and its neighboring regions, formed by a back-arc extensional tectonic event linked to the proto-Tethys Ocean subduction and seafloor spreading^58,60^. The Tarim block broke away from the northern side of the Australia block, and then drifted from the supercontinent Gondwana^61^. This model has recently been supported by Gao *et al*.^34^ based on study of the Taxkorgan region in West Kunlun. Ages for a metabasalt (516 Ma) and metadacite (521 Ma) in the Taaxi region likely represent depositional ages of bimodal volcanic rocks in the Early Cambrian, associated with the extensional event^34^ that led to the formation of a back-arc rift basin^27^. After the breakup of Gondwana, the Tarim block drifted northwest into the proto-Tethys Ocean, leading to a broad rift within the open ocean (Zhang *et al*.^35^, see their figure 14, and Li & Powell^61^, see their figure 7). In addition to the Taxkorgan Fe-rich basins studied herein, the Dahongliutan hematitic BIF^62^ has recently been discovered to the east of the Western Kunlun orogenic belt, south of the Tarim block (formational age: 593–532 Ma), while on the opposite side of the rift in northern Australia, Fe-rich chemical sediments were similarly deposited^63^. The temporal and spatial correspondence of these deposits indicates that the Fe-rich sediments were spatially widespread. Furthermore, although a large number of volcanogenic massive sulfide (VMS) deposits are present in western China (e.g., Qilian and Altai-Junggar), the closest known VMS deposit of early Paleozoic age is more than 2000 km from our study area, and significantly these VMS deposits are mainly younger than Late Cambrian^64^, thus suggesting that the Taxkorgan Fe-rich sedimentary rocks are unrelated to VMS systems in western China. Hence, similar to Archaean Algoma-type BIF that formed in back-arc basins^1^, the Lower Cambrian BIF examined here formed in a basin that was likely influenced by an enhanced hydrothermal iron flux, but critically, that marine conditions within that basin (i.e., persistent widespread ferruginous and anoxic waters) were fundamentally different than those in modern oceans.

Consistent with this view, the distribution of REE patterns in the Jiertieke BIF suggests that the basin was relatively broad and unlikely to have contained extremely stratified waters (e.g., like the modern Red Sea). Foremost, we have found significant positive Eu anomalies exist, the magnitude of these anomalies varies considerably within strata of the iron belts, suggesting multiple REE sources and significant REE scavenging. Specifically, the lack of Eu anomalies in the Yelike and Taaxi regions is inconsistent with the formation of the Fe deposits within a small, strongly stratified basin, where one would expect persistent strong positive Eu anomalies. We therefore suggest that records from this basin can provide a glimpse into redox evolution with a Cambrian marine basin, rather than simply representing an anomalous setting.

Based on the new geochronology data presented here, coupled with sedimentological, mineralogical, and geochemical data on the BIF, we provide evidence for the deposition of a series of large-scale, Early Cambrian Fe-rich chemical sediments. Based on differences in REE patterns, these Fe-rich sediments can be distinguished from modern Fe-exhalite deposits (e.g., lack of Eu anomalies in Taaxi and Yelike), and indicate a genesis similar to that proposed for Archean Algoma-type BIF^25^. The absence of true negative Ce anomalies in the chemical sedimentary rocks suggests that marine anoxia and ferruginous conditions extended well into the Early Cambrian, offering support for recent Fe-speciation studies^7,11^, and indicating that the Early Cambrian was characterized, at least locally, by stratified, redox-heterogeneous basins^65,66^.

## Methods

### Major elements

Major element oxide abundances for bulk iron-rich sedimentary rock samples were determined using an X-ray fluorescence spectrometer with an analytical error of less than 5% at ALS Chemex (Guangzhou, China). Loss on ignition (LOI) was determined by heating powders at 1000 °C for 2 hours. Decreased weights of the powders were then calculated.

### Trace elements

Samples were broken into chips, with any chips showing evidence of veins or secondary alteration removed, and the remaining sample was then ground to a fine powder in a tungsten steel grinding mill. About 50 mg of the powder was weighed, then dissolved in 1 mL concentrated HF and 1 mL 8 M HNO_3_ in a 15 mL Savillex^TM^ Teflon vial. The temperature was maintained at 130 °C for 48 hours. Following this step, 2 mL of 8 M HNO_3_ was added immediately before the solution was evaporated to dryness at 130 °C. Following evaporation, 2 mL of 8 M HNO_3_ was added to achieve complete dissolution of the sample. After the solution cooled, samples were transferred to polyethelene bottles containing 50 g 2% HNO_3_ and a 10 ng g^−1^ internal indium standard. Trace element concentrations were analyzed on an Element I Finnigan MAT inductively coupled plasma-mass spectrometer (ICP-MS) at the Institute of Geology and Geophysics, Chinese Academy of Sciences (IGGCAS), Beijing. Two standard samples, FER-1 and FER-2, were used to monitor analyses. The accuracy of measured concentrations is 1 to 5%.

### U-Pb zircon geochronology

The outcrop sample used for geochronology was taken from interlayered biotite-quartz schist occurring within Fe-rich sedimentary rocks. Conventional magnetic and density techniques were used to separate zircons from the sample. Zircons were then mounted in epoxy resin and polished to section the crystals for imaging and analysis. Transmitted and reflected light micrographs, as well as cathodoluminescence (CL) images, were taken using a CAMECA electron microprobe (EMPA) at IGGCAS. The CL images were used to reveal the internal structures and zoning of the zircons. Thorium and Pb isotopes were then analyzed on a Cameca 1280 secondary ion mass spectrometer (SIMS) at the IGGCAS. The operating procedure and subsequent calculations have been described previously in detail^67^. A common Pb correction was carried out based on the interference- and background-corrected ^204^Pb signal and a model Pb composition^68^. Uncertainties on individual analysis in the data are reported at the 1σ level; mean ages for U/Pb (and Pb/Pb) analyses are presented with a 95% confidence interval, and the data were plotted using ISOPLOT^69^.

## Electronic supplementary material

Supplementary Information and Figures
