# Supplementary material for: Earth’s youngest banded iron formation implies ferruginous conditions in the Early Cambrian ocean
Source: Sci Rep. 2018 Jul 2;8:9970. doi: 10.1038/s41598-018-28187-2 (PMC6028650; doi:10.1038/s41598-018-28187-2)
Supplement: Supplementary file 1 — Supplementary Information and Figures [file 41598_2018_28187_MOESM1_ESM.pdf]

Earth's youngest banded iron formation implies ferruginous conditions in the Early Cambrian ocean

Zhi-Quan Li[a,b,c], Lian-Chang Zhang[b*], Chun-Ji Xue[a*], Meng-Tian Zheng[b], Ming-Tian Zhu[b], Leslie J. Robbins[c], John F. Slack[d], Noah J. Planavsky[e], & Kurt O. Konhauser[c]

[a] State Key Laboratory of Geological Processes and Mineral Resources, China University of Geosciences, Beijing 100083, China

[b] Key Laboratory of Mineral Resources, Institute of Geology and Geophysics, Chinese Academy of Sciences, Beijing 100029, China

[c] Department of Earth and Atmospheric Sciences, University of Alberta, Edmonton, Alberta T6G 2E3, Canada

[d] U.S. Geological Survey, National Center, MS 954, Reston, Virginia 20192, USA

[e] Department of Geology and Geophysics, Yale University, New Haven, Connecticut 06520, USA

Correspondence to L.-C.Z. (email: lczhang@mail.iggcas.ac.cn) or C.-J.X. (email: chunji.xue@cugb.edu.cn).

Supplementary information

Regional geology

The West Kunlun Orogenic Belt is south of the Tarim block, and north of the Karakorum Terrane, with the Oytag-Kegang and Karakorum-Longmucuo-Shuanghu Faults constraining the eastern and western boundaries, respectively. There are two suture zones, oriented from north to south, in the West Kunlun Orogenic Belt: the Kudi and Kangxiwa Faults[1]. Based on these structural boundaries, the West Kunlun Orogenic Belt may be subdivided into three different tectonic units: the North Kunlun, the South Kunlun, and the Taxkorgan-Tianshuihai Terrane.

The Taxkorgan terrane is located in the South of the West Kunlun orogenic belt, and is bounded by the Kangxiwa Fault to the north, and the Taaxi Fault in the Karakorum fault zone to the south. The faults are oriented NW-SE in the Taxkorgan region, with the field area being dominated by the previously defined Precambrian Bulunkuole Complex and early Cretaceous Xialafudi Group.

The Bulunkuole Group is distributed with an area of 200 km long and 5-50 km wide in N-S direction[2]. This group was intruded by the Triassic Kongur granodiorite-granite pluton (ca. 1200 km^2) in the west and by the early Paleozoic Datong granite pluton (ca. 800 km^2) in the east[3,4]. The Bulunkuole Group in the Taxkorgan region is characterized by a suit of volcanic-sedimentary rocks, which are also the main host for the iron deposits in this section. The strata strike to NW 358°-45°, with moderate to steep dips ranging from 20° to 75°. The thickness of the Bulunkuole Group is 14,700 m, from bottom to top can be subdivided into three

sections: Section 1 is mainly composed of occurrences of biotite-quartz schist, biotite hornblende-quartz schist, biotite hornblende-plagioclase schist, quartzite, and minor marble, with a thickness of 3400 m. The 4300 m thick section 2 is mainly comprised of biotite-quartz schist, metavolcanics and quartzite; the quartzite is interlayered with biotite-quartz schist. Section 3 mainly present in the northeast of the Bulunkuole Group, and comprised of quartzite interlayered with biotite-quartz schist at bottom and biotite-quartz schist, quartz schist, biotite-plagioclass schist, metavolcanics, and marble at top, and is 4700 m thick. A geological survey revealed that the Bulunkuole Group is dominated by a volcanic-sedimentary sequence and volcanic rocks (metadacite and metabasalt) that account for ~ 30%, whereas sedimentary strata account for ~70%, with respect to the thickness. Most outcrops preserve original sedimentary bedding, and are comprised of biotite-quartz schist, quartzite, hornblende-quartz schist, amphibolite, and minor dolomitic marble. The Fe-rich sediments mostly occur within biotite-quartz schist, or between metabasalt and metadacite. These Fe-rich sediments mainly are dominantly quartz-magnetite and biotite-quartz magnetite.

Supplementary figures

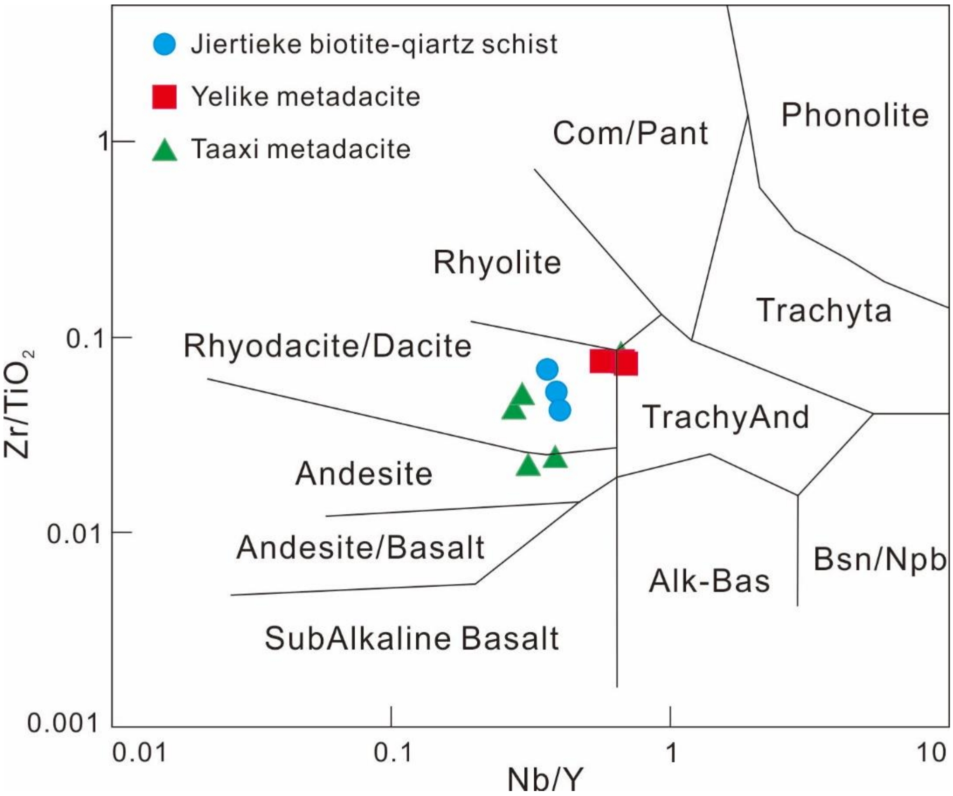

Fig. S1 Zr/TiO_2-Nb/Y diagram[5] for the Jiertieke biotite-quartz schist, and metadacite form Yelike and Taaxi, indicating the source of the schist relative to the metadacite in Yelike and Taaxi.

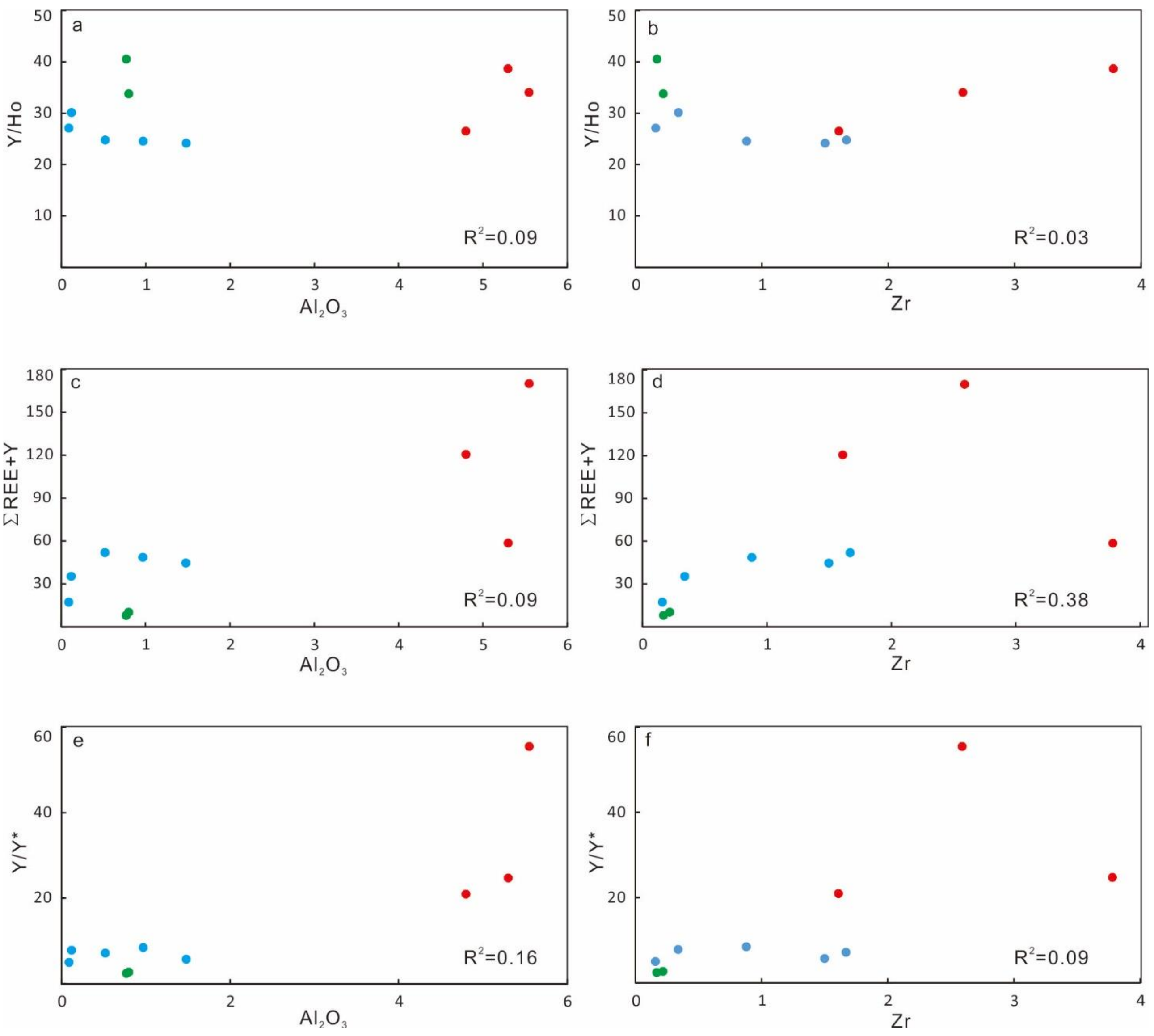

Fig. S2 Cross plots of (a) Al_2O_3 (wt. %) vs Y/Ho ratios; (b) Zr (ppm) vs Y/Ho ratios; (c) Al_2O_3 (wt. %) vs ∑REE+Y (ppm); (d) Zr (ppm) vs ∑REE+Y (ppm); (e) Al_2O_3 (wt. %) vs Y/Y*; and (f) Zr (ppm) vs Y/Y* for the Jiertieke BIF. Collectively, these cross plots indicate the contamination of the initial chemical precipitates with a minor detrital component, as well as the overprinting of Y/Ho ratios by a near-chondritic hydrothermal source.

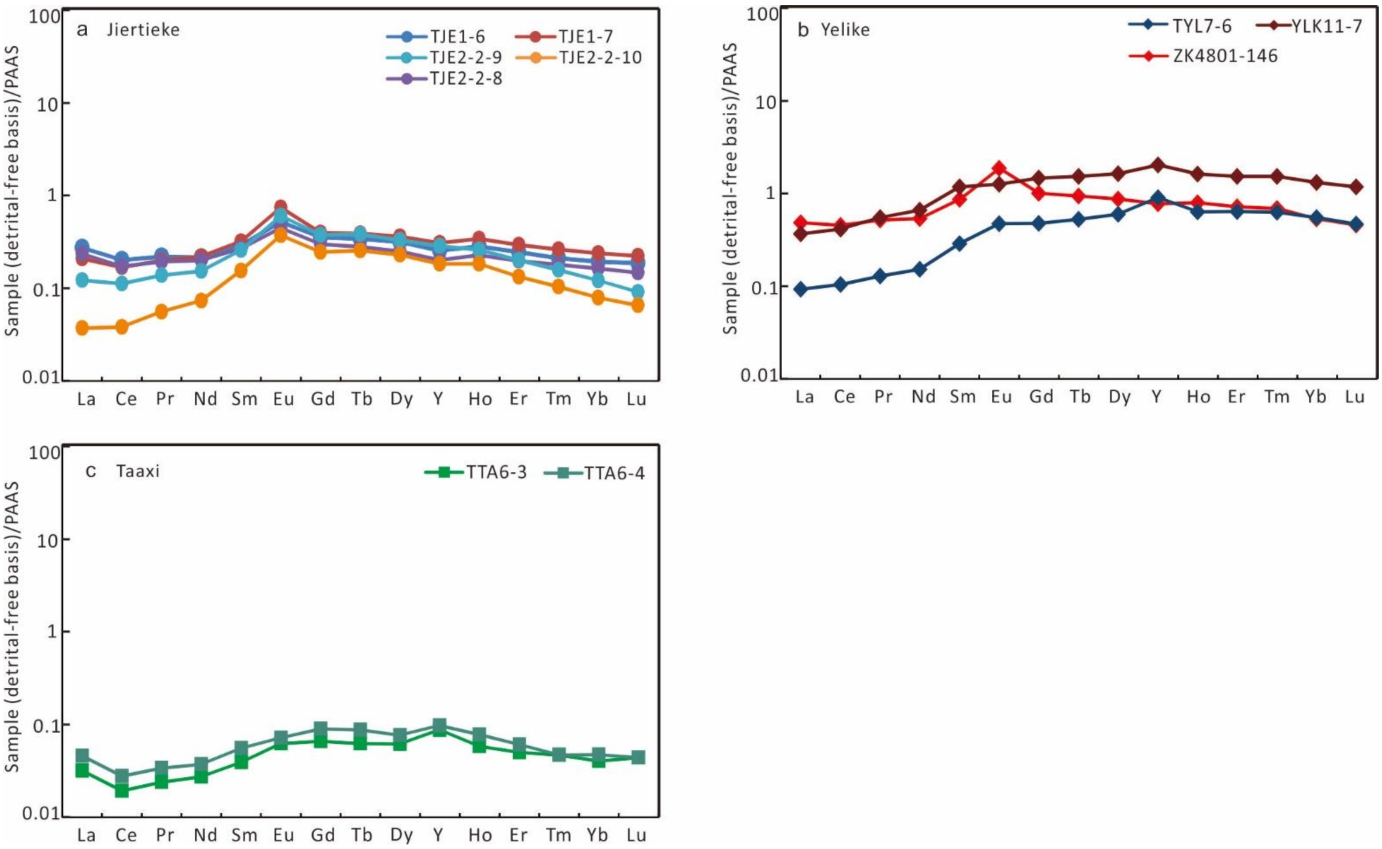

Fig. S3 REE systematics of the BIF from the (a) Jiertieke, (b) Yelike, and (c) Taaxi regions. REE data are re-calculated on a detrital-free basis, using PAAS as the detrital component and Hf as the normalizing element, and correspond well to the REE patterns of uncorrected bulk samples.

The simple formulas used are:

Detrital component = $Hf_{(sample)}/Hf_{(PAAS)}$

Detrital-free = $Element_{(Sample)} - Element_{(PAAS)} \times$ Detrital $component_{(Sample)}$

References

1. Pan, Y., Wang, Y., Matte, Ph., Tapponnier, P. Tectonic evolution along the geotraverse from Yecheng to Shiquanhe. In *Chinese Science Abstracts Series B* **68**, 295–307 (1994). (in Chinese with English abstract)

2. Zhang, C.L., Zou, H.B., Ye, X.T. & Chen, X.Y. Tectonic evolution of the NE section of the Pamir Plateau: New evidence from field observations and zircon U-Pb geochronology. *Tectonophysics* **723**, 27-40 (2018).

3. Jiang, Y.H., Rui, X.J., He, J.R., Guo, K.Y. & Yang, W.Z. Tectonic type of Caledonian granitoids and tectonic significance in the west Kunlun Mts. *Acta Petrologica Sinica* **15**, 105–115 (1999). (in Chinese with English abstract).

4. Jiang, Y.H. *et al*. Petrology and geochemistry of shoshonitic plutons from the western Kunlun orogenic belt, northwestern Xinjiang, China: implications for granitoid geneses. *Lithos* **63**, 165–187 (2002).

5. Winchester, J.A. & Floyd, P.A. Geochemical discrimination of different magma series and their differentiation products using immobile elements. *Chemical geology* ***20***, 325-343 (1977).

Supplementary tables

Table S1. Major oxide (wt.%) and trace element (ppm) compositions of the biotite-quartz schist in the Jiertieke, and metadacite in the Yelike, Taaxi area.

Citation	This article			Zheng, 2017 (Ref. 27)							
Location	Jiertieke (biotite-quartz schist)			Taaxi (metadacite)					Yelike (metadacite)		
Sample	TJE1-1	TJE1-2	TJE1-3	TTA1-1	TTA1-2	TTA1-4	TTA1-5	TTA1-21	ZK4001-59	TYL6-8	ZK4001-52
SiO_2	65.00	67.82	64.84	64.48	75.33	68.51	67.47	69.72	72.02	72.88	71.73
TiO_2	0.54	0.41	0.39	0.72	0.25	0.39	0.43	0.61	0.34	0.34	0.33
Al_2O_3	14.35	13.21	13.25	14.31	12.15	12.10	12.33	15.03	14.57	14.14	14.59
TFe_2O_3	6.53	4.75	6.70	6.94	1.98	2.97	3.48	4.68	3.28	3.17	3.25
MnO	0.24	0.19	0.22	0.08	0.06	0.16	0.18	0.05	0.01	0.01	0.02
MgO	4.26	3.57	4.45	3.42	0.44	0.83	0.99	1.01	0.83	1.06	0.58
CaO	3.63	3.88	3.87	1.33	3.40	3.27	3.32	1.04	0.50	0.41	0.95
Na_2O	0.45	1.89	0.76	7.50	4.85	1.34	1.44	8.08	7.43	7.04	7.04

K_2O	4.02	3.67	4.61	0.21	0.12	7.48	7.42	0.32	0.48	0.08	0.52
P_2O_5	0.16	0.13	0.12	0.20	0.04	0.08	0.08	0.20	0.07	0.09	0.05
LOI	0.18	0.24	0.22	0.62	1.32	2.40	2.50	0.08	0.50	0.56	0.54
TOTAL	99.36	99.76	99.43	99.81	99.94	99.69	99.79	100.82	100.03	99.78	99.60
Sc	11.55	10.33	11.44	13.53	5.89	12.67	13.14	6.15	2.20	4.25	5.69
Co	2.86	1.93	4.63	3.30	4.29	3.73	3.73	2.67	6.35	11.68	7.67
Ni	1.74	0.67	4.01	10.47	13.82	6.14	7.16	42.35	186.41	34.91	8.36
Cu	8.13	0.95	11.07	7.63	4.54	2.55	2.54	14.89	76.06	14.99	5.11
Zn	111.27	70.71	116.57	30.02	7.60	13.69	14.99	15.11	17.55	18.99	14.93
Ga	14.95	14.36	15.42	19.17	12.20	13.88	14.55	15.17	19.12	17.83	19.05
Rb	124.63	118.51	133.74	4.93	0.50	107.34	110.92	9.04	15.36	1.37	18.68
Sr	26.11	57.98	33.04	41.27	68.17	63.05	60.90	46.30	39.10	35.21	51.00
Y	30.29	31.60	36.47	24.80	16.02	31.87	33.03	17.88	16.71	19.92	15.34
Zr	230.07	215.03	266.66	167.85	198.70	190.71	178.85	129.26	247.60	256.00	247.77

Nb	12.17	12.36	13.19	9.59	10.70	9.40	9.09	5.54	11.61	11.40	10.37
Cs	2.91	4.53	5.05	0.39	0.09	0.53	0.34	0.29	0.82	0.35	0.91
Ba	246.94	399.83	231.09	90.70	25.70	1497.80	1463.20	99.90	87.90	13.70	127.90
La	34.45	36.98	41.59	21.31	11.59	14.86	14.76	9.61	5.28	6.79	16.37
Ce	60.91	67.82	76.66	44.17	27.39	29.73	29.66	24.95	11.40	18.78	32.11
Pr	7.95	8.37	9.55	6.15	3.48	3.94	3.97	3.06	1.24	2.03	3.51
Nd	30.74	33.08	37.29	26.22	14.10	16.12	16.39	13.81	4.85	9.41	12.77
Sm	5.87	6.34	7.05	6.40	2.57	3.68	3.70	3.20	1.44	2.58	2.94
Eu	1.15	1.25	1.40	1.71	0.38	0.67	0.68	0.97	0.51	0.74	0.94
Gd	5.42	5.93	6.58	6.30	2.13	4.19	4.33	3.16	1.92	3.00	2.78
Tb	0.91	0.99	1.13	0.99	0.38	0.83	0.85	0.53	0.41	0.55	0.43
Dy	5.24	5.92	6.70	5.41	2.43	5.59	5.75	3.34	2.98	3.52	2.58
Ho	1.10	1.21	1.36	1.05	0.58	1.30	1.28	0.69	0.65	0.75	0.53
Er	3.27	3.34	3.91	2.62	1.91	3.47	3.64	1.96	1.91	2.24	1.58

Tm	0.51	0.50	0.59	0.38	0.35	0.52	0.54	0.31	0.29	0.34	0.26
Yb	3.40	3.28	3.83	2.61	2.57	3.42	3.59	1.97	2.00	2.34	1.77
Lu	0.55	0.54	0.61	0.46	0.43	0.55	0.56	0.31	0.31	0.38	0.30
Hf	6.73	6.50	7.52	5.21	5.85	5.37	5.07	3.87	7.22	7.53	7.14
Ta	0.95	1.00	1.03	0.48	0.53	0.63	0.64	0.41	0.96	0.95	0.89
Tl	0.75	1.23	1.08	0.05	0.04	0.19	0.19	0.05	0.14	0.05	0.15
Pb	3.07	1.97	2.83	1.89	4.12	1.84	1.41	1.23	4.55	2.47	2.56
Bi	0.09	0.01	0.05	0.02	0.07	0.00	0.00	0.01	0.02	0.05	0.03
Th	12.33	11.67	12.89	4.10	9.61	6.48	6.61	3.87	7.68	12.14	9.65
U	2.38	2.55	2.79	0.81	1.15	1.62	1.59	1.14	1.61	2.07	1.71
∑REE+Y	191.75	207.14	234.72	150.58	86.31	120.74	122.73	85.75	51.90	73.37	94.21
Th/Sc	1.07	1.13	1.13	0.30	1.63	0.51	0.50	0.63	3.49	2.86	1.70

Table S2. Major oxide (wt.%) and trace element (ppm) compositions of the BIFs in the Jiertieke, Yelike, Taaxi area. Coupled with comparition data

Citation	This article					Ref. 27					Ref. 73	Ref. 71	Ref. 46	Ref. 72	Ref. 74	Ref. 49	
Location	Jiertieke area					Yelike area			Taaxi area		Um Anab (Egypt)	Biwabik BIF(U.S)	Jerome(U.S)	Raptain (Canada)	Atlantis II Deep(Red Sea)	Loihi Seamount (Hawaii)	
Sample	TJE1-6	TJE1-7	TJE2-2-8	TJE2-2-9	TJE2-2-10	ZK4801-146	TYL7-6	YLK11-7	TTA6-3	TTA6-4	AVERAGE	AVERAGE (BIF+SIF)	AVERAGE (Iron FM)	AVERAGE (hem-jasp)	AVERAGE (Fe-rich sediments)	AVERAGE (Fe-oxyhydroxide)	Mn-rich Fe-oxyhydroxide
SiO_2	54.10	19.95	25.20	21.30	23.50	48.28	56.32	37.37	24.63	18.01	54.62	-	18.16	-	11.26	16.81	18.94
TiO_2	0.06	0.07	0.10	0.01	0.01	0.20	0.16	0.28	0.03	0.02	0.20	-	0.04	-	0.06	0.20	0.42
Al_2O_3	0.52	0.97	1.48	0.12	0.09	4.80	5.30	5.55	0.77	0.80	3.60	-	1.04	-	1.13	0.89	2.18
MnO	0.19	1.05	0.83	0.70	0.63	0.05	0.01	0.19	0.14	0.17	0.07	-	0.13	-	-	0.01	6.60
MgO	0.74	6.77	7.16	1.44	1.50	2.84	0.11	4.07	1.25	1.54	1.28	-	0.09	-	-	1.17	2.65
CaO	1.70	14.15	15.20	8.14	5.57	5.75	0.41	2.66	0.80	1.06	2.98	-	0.92	-	5.04	0.89	2.18
Na_2O	0.30	0.43	0.74	0.02	0.02	2.67	3.01	1.67	0.12	0.08	0.25	-	0.09	-	-	4.50	4.82
K_2O	0.00	0.01	0.02	0.00	0.00	0.60	0.02	0.65	0.02	0.02	0.12	-	0.22	-	-	0.52	1.05
P_2O_5	0.03	0.03	0.05	0.06	0.04	0.10	0.13	0.16	0.03	0.04	0.54	-	0.54	-	0.28	1.31	2.45
$\sum FeO$	41.99	48.48	39.77	64.18	66.55	29.03	30.06	46.34	71.73	77.77	35.14	-	77.78	-	47.33	50.84	36.82
LOI	0.20	7.98	9.35	3.88	1.93	5.11	3.90	0.54	0.10	0.10	0.53	-	0.58	-	-	22.80	19.29

Total	99.83	99.89	99.90	99.86	99.84	99.43	99.43	99.48	99.61	99.61	99.33	-	99.57	-	-	100.22	98.80
FeO	11.45	14.50	11.80	18.75	19.30	-	-	-	-	-	-	-	-	-	-	-	-
SiO_2+∑FeO	96.09	68.43	64.97	85.48	90.05	77.31	86.38	83.71	96.36	95.78	-	-	-	-	-	-	-
TiO_2+Al_2O_3	0.58	1.04	1.58	0.13	0.10	5.00	5.46	5.83	0.80	0.82	-	-	-	-	-	-	-
Rb	3.52	0.02	0.38	0.14	0.01	151.40	1.03	19.63	0.01	0.04	-	-	-	-	-	-	-
Ba	116.60	29.27	44.03	12.06	16.92	262.41	28.95	237.52	2.63	5.44	-	15.99	-	-	-	-	-
Sc	2.56	2.08	2.36	0.17	0.04	9.71	8.20	9.07	0.46	0.56	-	-	-	-	-	-	-
Th	2.43	1.91	2.46	0.22	0.14	5.90	3.41	6.93	0.09	0.14	-	-	-	-	0.43	-	-
U	3.70	2.17	3.08	1.69	0.82	1.80	0.95	0.84	0.66	0.96	-	-	-	-	9.51	-	-
Ta	0.18	0.12	0.15	0.02	0.01	0.39	0.27	0.30	0.02	0.02	-	-	-	-	-	-	-
Pb	7.49	3.06	4.08	1.62	1.74	2.42	1.95	1.43	0.27	0.41	-	-	-	-	181.16	-	-
Sr	73.97	54.17	57.87	146.20	92.15	422.46	27.61	40.29	10.35	9.83	-	-	-	-	1.26	-	-
Nb	2.26	1.50	1.89	0.16	0.13	4.26	4.92	1.75	0.21	0.30	-	-	-	-	-	-	-
Zr	1.67	0.88	1.50	0.34	0.16	1.61	3.78	2.59	0.17	0.22	-	11.56	-	-	7.90	-	-
Hf	0.05	0.03	0.05	0.02	0.01	0.06	0.11	0.20	0.01	0.01	-	-	-	-	<0.5	-	-
Sr/Ba	0.63	1.85	1.31	12.12	5.45	1.61	0.95	0.17	3.94	1.81	-	-	-	-	-	-	-
Th/U	0.66	0.88	0.80	0.13	0.17	3.28	3.59	8.25	0.14	0.15	-	-	-	-	0.05	-	-

Zr/Hf	33.40	29.33	30.00	17.00	16.00	26.83	34.36	12.95	17.00	22.00	-	-	-	-	-	-	-
Hf/Ta	0.28	0.25	0.33	1.00	1.00	0.15	0.41	0.67	0.50	0.50	-	-	-	-	-	-	-
Th/Sc	0.95	0.92	1.04	1.29	3.50	0.61	0.42	0.76	0.20	0.25	-	-	-	-	-	-	-
La	10.81	8.22	9.35	4.80	1.49	18.78	4.35	15.37	1.30	1.84	12.13	2.75	8.06	14.24	3.62	2.87	3.20
Ce	16.86	13.88	14.41	9.18	3.19	36.67	9.97	35.68	1.70	2.38	27.09	5.86	21.58	34.89	7.46	5.02	2.35
Pr	2.03	1.81	1.79	1.25	0.51	4.63	1.32	5.14	0.23	0.32	3.78	0.67	1.85	4.59	0.84	0.63	0.63
Nd	7.64	7.62	7.06	5.32	2.55	18.32	5.83	23.42	1.00	1.34	16.58	2.68	7.25	20.30	3.25	2.75	2.73
Sm	1.66	1.81	1.54	1.44	0.87	4.80	1.70	6.67	0.23	0.32	3.98	0.56	1.58	4.65	0.64	0.63	0.59
Eu	0.57	0.81	0.49	0.65	0.40	2.01	0.53	1.39	0.07	0.08	1.05	0.18	0.46	1.13	0.43	0.27	0.19
Gd	1.70	1.89	1.43	1.77	1.15	4.70	2.29	6.93	0.32	0.43	4.65	0.58	1.99	5.43	0.61	0.75	0.56
Tb	0.27	0.31	0.22	0.30	0.20	0.73	0.42	1.20	0.05	0.07	0.75	0.09	0.36	0.86	0.09	0.12	0.11
Dy	1.53	1.71	1.22	1.56	1.08	4.09	2.86	7.73	0.30	0.37	4.67	0.57	2.26	5.82	0.53	0.73	0.77
Y	7.16	8.46	5.69	7.83	4.98	20.93	24.72	55.44	2.43	2.70	26.60	3.53	16.93	37.50	2.88	4.14	7.00
Ho	0.29	0.35	0.24	0.26	0.18	0.79	0.64	1.63	0.06	0.08	1.06	0.12	0.47	1.25	0.10	0.14	0.18
Er	0.73	0.85	0.59	0.58	0.38	2.05	1.86	4.43	0.15	0.18	3.19	0.36	1.35	3.72	0.28	0.40	0.50
Tm	0.09	0.11	0.08	0.07	0.04	0.28	0.26	0.63	0.02	0.02	0.47	0.05	0.18	0.54	0.04	0.06	0.07
Yb	0.57	0.69	0.49	0.35	0.23	1.51	1.59	3.77	0.12	0.14	3.19	0.31	1.01	3.52	0.26	0.36	0.42
Lu	0.09	0.10	0.07	0.04	0.03	0.20	0.21	0.52	0.02	0.02	0.50	0.04	0.13	0.52	0.04	0.06	0.06

∑REE+Y	51.98	48.60	44.66	35.39	17.29	120.49	58.54	169.94	8.01	10.28	109.69	18.36	65.48	138.96	18.18	18.93	19.36
$(La/Yb)_{PAAS}$	1.39	0.88	1.41	1.01	0.48	0.92	0.20	0.30	0.80	0.97	0.38	0.66	0.80	0.40	1.03	0.59	0.56
La/La*	1.19	1.29	1.27	1.13	1.74	1.00	1.09	1.10	1.78	1.62	1.04	1.06	1.06	1.03	1.00	1.49	1.58
Ce/Ce*	0.90	0.94	0.92	0.91	1.00	0.91	0.99	0.95	0.95	0.91	0.93	1.03	1.33	0.99	0.99	1.04	0.47
Eu/Eu*	1.66	2.13	1.61	2.00	1.96	2.09	1.28	0.98	1.32	1.08	1.22	1.59	1.26	1.13	3.42	1.96	1.49
Y/Y*	0.86	0.88	0.85	0.97	0.89	0.93	1.46	1.25	1.44	1.25	0.95	1.06	1.31	1.11	0.98	1.02	1.50
Pr/Pr*	1.05	1.03	1.04	1.04	1.00	1.05	1.01	1.02	1.02	1.04	1.03	0.99	0.86	1.00	1.01	0.98	1.30
Y/Ho	24.76	24.53	24.11	30.12	27.09	26.49	38.63	34.01	40.50	33.75	25.07	28.44	35.83	30.00	27.67	28.75	38.89
Sm/Yb	2.89	2.63	3.15	4.12	3.79	3.18	1.07	1.77	1.92	2.29	1.25	1.81	1.56	1.32	2.48	1.75	1.50

Formula: $La/La^* = La_{PAAS}/(3xPr_{PAAS}-2Nd_{PAAS})$; $Ce/Ce^* = 2xCe_{PAAS}/(La_{PAAS}+Pr_{PAAS})$; $Pr/Pr^* = 2xPr_{PAAS}/(Ce_{PAAS}+Nd_{PAAS})$;

$Eu/Eu^* = Eu_{PAAS}/(0.67xSm_{PAAS}+0.33xTb_{PAAS})$; $Y/Y^* = 2xY_{PAAS}/(Dy_{PAAS}+Ho_{PAAS})$

Table S3 Zircon U-Pb dating results of biotite-quartz schist (sample 15TJE1-1) in the Jiertieke area

	U(ppm)	Th(ppm)	Pb(ppm)	Th/U	f_{206}%	$^{207}Pb/^{235}U$	±σ(%)	$^{206}Pb/^{238}U$	±σ(%)	$^{207}Pb/^{206}Pb$	±σ(Ma)	$^{207}Pb/^{235}U$	±σ(Ma)	$^{206}Pb/^{238}U$	±σ(Ma)
15TJE1-1@04	117	100	12	0.85	1.29	0.59942	3.29	0.0769	1.50	474.4	63.6	476.9	12.6	477.4	6.9
15TJE1-1@01	364	362	39	0.99	0.37	0.63095	1.85	0.0793	1.50	519.0	23.4	496.7	7.3	491.9	7.1
15TJE1-1@13	111	66	11	0.60	1.11	0.60962	3.20	0.0808	1.52	401.3	61.9	483.3	12.4	500.8	7.3
15TJE1-1@03	89	60	9	0.67	0.56	0.66283	3.03	0.0828	1.51	531.1	56.5	516.3	12.3	513.0	7.4
15TJE1-1@15	283	216	30	0.76	0.15	0.65501	1.82	0.0831	1.50	499.2	22.3	511.6	7.3	514.3	7.4
15TJE1-1@24	158	115	17	0.73	0.15	0.66589	2.45	0.0833	1.50	528.7	41.9	518.2	10.0	515.8	7.5
15TJE1-1@19	222	199	25	0.90	0.10	0.67531	1.78	0.0844	1.50	532.0	20.8	523.9	7.3	522.1	7.5
15TJE1-1@12	100	62	11	0.62	0.10	0.66765	2.21	0.0844	1.51	505.2	35.1	519.3	9.0	522.5	7.6
15TJE1-1@14	518	427	57	0.82	0.10	0.68215	1.67	0.0846	1.51	547.5	15.5	528.1	6.9	523.6	7.6
15TJE1-1@10	244	215	28	0.88	0.09	0.67911	1.82	0.0847	1.52	534.8	21.7	526.2	7.5	524.3	7.7
15TJE1-1@25	100	64	11	0.64	0.21	0.68625	2.07	0.0848	1.50	555.7	30.8	530.5	8.6	524.7	7.6
15TJE1-1@18	147	133	17	0.90	0.02	0.65704	1.91	0.0849	1.50	457.7	26.0	512.8	7.7	525.2	7.6

15TJE1-1@06	299	170	31	0.57	0.14	0.67934	1.78	0.0853	1.50	520.7	20.9	526.4	7.3	527.7	7.6
15TJE1-1@17	141	128	16	0.91	0.15	0.69005	2.22	0.0860	1.52	536.8	35.1	532.8	9.2	531.9	7.7
15TJE1-1@22	282	224	31	0.79	1.36	0.65868	2.61	0.0860	1.56	433.8	45.8	513.8	10.6	532.0	8.0
15TJE1-1@11	242	230	28	0.95	0.12	0.69295	1.76	0.0861	1.51	543.2	19.6	534.6	7.3	532.5	7.7
15TJE1-1@09	766	772	91	1.01	0.08	0.68950	1.62	0.0863	1.50	526.9	13.2	532.5	6.7	533.8	7.7
15TJE1-1@16	309	343	38	1.11	0.11	0.69013	1.97	0.0869	1.50	515.5	27.6	532.9	8.2	536.9	7.8
15TJE1-1@08	426	441	51	1.04	0.23	0.70592	1.73	0.0873	1.50	555.2	18.8	542.3	7.3	539.3	7.8
15TJE1-1@21	101	96	12	0.95	0.29	0.69783	2.06	0.0874	1.50	526.7	30.6	537.5	8.6	540.0	7.8
15TJE1-1@20	279	243	33	0.87	0.07	0.70329	1.72	0.0886	1.50	512.5	18.4	540.8	7.2	547.5	7.9
15TJE1-1@23	366	224	42	0.61	0.15	0.73868	1.83	0.0917	1.52	544.7	22.3	561.6	7.9	565.8	8.2

Table S4. REE data calculated on detrital-free basis

	TJE1-6	TJE1-7	TJE2-2-8	TJE2-2-9	TJE2-2-10	ZK4801-146	TYL7-6	YLK11-7	TTA6-3	TTA6-4
La	10.43	7.99	8.96	4.64	1.42	18.32	3.51	13.84	1.22	1.76
Ce	16.06	13.40	13.62	8.86	3.03	35.71	8.22	32.50	1.54	2.22
Pr	1.94	1.76	1.70	1.21	0.49	4.52	1.13	4.79	0.21	0.30
Nd	7.30	7.41	6.72	5.18	2.48	17.91	5.08	22.06	0.93	1.27
Sm	1.60	1.78	1.48	1.42	0.86	4.73	1.58	6.45	0.22	0.31
Eu	0.56	0.80	0.48	0.65	0.40	2.00	0.51	1.35	0.07	0.08
Gd	1.66	1.86	1.39	1.75	1.14	4.64	2.19	6.74	0.31	0.42
Tb	0.26	0.30	0.22	0.29	0.20	0.72	0.40	1.17	0.05	0.07
Dy	1.49	1.69	1.17	1.54	1.07	4.03	2.76	7.54	0.29	0.36
Y	6.89	8.30	5.42	7.72	4.93	20.61	24.13	54.36	2.38	2.65
Ho	0.28	0.34	0.23	0.26	0.18	0.78	0.62	1.59	0.06	0.08
Er	0.70	0.83	0.56	0.57	0.38	2.02	1.80	4.32	0.14	0.17
Tm	0.09	0.11	0.07	0.06	0.04	0.28	0.25	0.61	0.02	0.02
Yb	0.54	0.67	0.46	0.34	0.22	1.48	1.53	3.66	0.11	0.13
Lu	0.08	0.10	0.06	0.04	0.03	0.19	0.20	0.50	0.02	0.02
$(La/Yb)_{PAAS}$	1.42	0.88	1.44	1.01	0.47	0.92	0.17	0.28	0.79	0.97
La/La*	1.19	1.30	1.29	1.14	1.81	1.00	1.11	1.12	1.87	1.67
Ce/Ce*	0.90	0.93	0.91	0.91	1.00	0.90	0.98	0.94	0.94	0.90
Eu/Eu*	1.68	2.15	1.63	2.02	1.97	2.10	1.29	0.98	1.33	1.08
Y/Y*	0.85	0.88	0.84	0.97	0.89	0.93	1.47	1.25	1.46	1.26
Pr/Pr*	1.05	1.03	1.04	1.04	1.00	1.05	1.01	1.02	1.03	1.05

Detrital_component = Hf(sample)/Hf(PAAS) ; Detrital-free = Element(Sample) - Element(PAAS) x Detrital_component(Sample); Formula: La/La* = $La_{PAAS}/(3xPr_{PAAS}-2Nd_{PAAS})$; Ce/Ce* = $2xCe_{PAAS}/(La_{PAAS}+Pr_{PAAS})$; Pr/Pr* = $2xPr_{PAAS}/(Ce_{PAAS}+Nd_{PAAS})$; Eu/Eu* = $Eu_{PAAS}/(0.67xSm_{PAAS}+0.33xTb_{PAAS})$; Y/Y* = $2xY_{PAAS}/(Dy_{PAAS}+Ho_{PAAS})$
